# Ivermectin Resistance in *Onchocerca volvulus:* Toward a Genetic Basis

**DOI:** 10.1371/journal.pntd.0000076

**Published:** 2007-08-30

**Authors:** Sara Lustigman, James P. McCarter

**Affiliations:** 1 Laboratory of Molecular Parasitology, Lindsley F. Kimball Research Institute, New York Blood Center, New York, New York, United States of America; 2 Divergence Inc., St. Louis, Missouri, United States of America; 3 Department of Genetics, Washington University School of Medicine, St. Louis, Missouri, United States of America; PLoS, United States of America

## Background

Onchocerciasis (river blindness) is a human disease caused by the filarial worm *Onchocerca volvulus.* Adult worms can live for over a decade in skin nodules of affected humans, releasing millions of microfilariae that cause debilitating itching and blindness [1]. An estimated 37 million people are infected [2], and there are 46,000 new cases of blindness annually (http://www.apoc.bf/).

**Figure 1:**
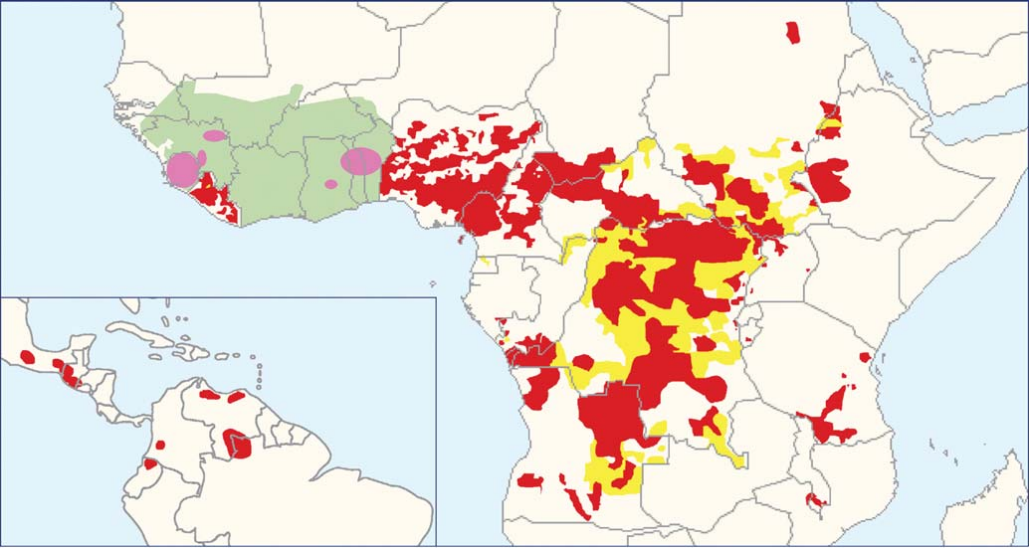
Distribution of Onchocerciasis Showing Current Status of Global Onchocerciasis Control. Red shading represents areas receiving ivermectin treatment. Yellow shading represents areas requiring further epidemiological surveys. Green shading indicates the area covered by the OCP in West Africa. Pink zones indicate the special intervention zones, i.e., previous OCP areas receiving ivermectin and some vector control. Figure from [10].

International programs supported by the World Health Organization and many other groups have worked to control the impact of onchocerciasis using vector control with insecticides beginning in 1974 and mass drug administration (MDA) with ivermectin (IVM, brand name Mectizan) beginning in 1987 (Figure 1) [3]. IVM is a highly effective microfilaricide and inhibits female worm microfilarial production for several months. Annual IVM MDA reduces morbidity [4,5] and lowers transmission [6,7]. From 1974 to 2002, the Onchocerciasis Control Programme (OCP) in West Africa greatly decreased *O. volvulus* transmission in the 11 OCP countries and prevented 600,000 cases of blindness [8–10]. IVM without vector control has been the principal tool for the Onchocerciasis Elimination Program of the Americas (1992–present) [9] and the African Programme for Onchocerciasis Control (1995–present). In the Americas, where *O. volvulus* is less common, the Onchocerciasis Elimination Program has substantially reduced transmission and is on track to eliminate the disease [9].

The African Programme for Onchocerciasis Control has extended treatment to 19 countries beyond those originally included in the OCP through sustainable community-directed IVM treatment [1,11]. By the end of 2005, 400 million treatments had been supplied in Africa by Merck’s Mectizan Donation Program, with an estimated 40 million people treated by nearly 300,000 community distributors (http://www.apoc.bf/). Nevertheless, the ecology of the disease in Africa, including the broad geographic range of *O. volvulus* and its blackfly vector, leads to the estimation that IVM treatment of at least 65% of the population for 25 or more years will be necessary to eliminate infection [9,12]. There are significant logistical obstacles to achieving such broad-ranging and prolonged treatment, and there is also concern that *O. volvulus* resistance to IVM will emerge. IVM resistance has become widespread in many parasitic nematodes of livestock [13,14]. At present there are no alternative drugs for IVM for use in the *Onchocerca* MDA programs that reduce microfilariae or kill adult worms, which can live up to 15 years in the human host.

The emergence of drug-resistant *O. volvulus* has been suggested by reports of patients failing to respond to IVM treatment [15,16]. A recent report from Ghana has provided the first proof of IVM resistance in *O. volvulus:* Mike Osei-Atweneboana and colleagues showed that the ability of IVM to suppress skin microfilariae repopulation was reduced in some communities that had received 6–18 years of IVM MDA [17]. The authors predict that a high rate of repopulation of skin with microfilariae will allow continued parasite transmission, possibly with IVM-resistant *O. volvulus* leading to disease recrudescence. Additionally, studies have associated IVM resistance with genetic markers [18–25], particularly the β*-tubulin* gene in human *O. volvulus* and the livestock nematode parasite *Haemonchus contortus* [22,26]. However, previous *O. volvulus* genotyping studies were non-longitudinal, using worms collected from different IVM-naïve and treated individuals.

## A New Study: IVM Causes Genetic Selection on *O. volvulus*

A new study by Catherine Bourguinat and colleagues published in *PLoS Neglected Tropical Diseases* extends these previous reports and concludes not only that IVM causes genetic selection on *O. volvulus* worms, but that this selection is also associated with a lower reproductive rate of the female parasites [27]. In this study of *O. volvulus* treatment in a hyperendemic region of central Cameroon, parasite genotypes (β-*tubulin* gene and two controls) and phenotypes (female fertility) were characterized in worms collected from the same individuals before and after four or 13 IVM treatments over three years. Parasites were collected pre- and post-treatment from clinical trial patients in four IVM treatment groups: 150 µg/kg of body weight annually or three-monthly, and 800 µg/kg annually or three-monthly.

Analyses of the genetic polymorphism in parasites pre- and post- treatment clearly showed a significant selection for β-*tubulin* heterozygotes in female worms. The most marked effect was in the three-monthly treated groups, where the frequency of the β-*tubulin* “aa” homozygotes post-IVM was reduced on average from 68.6% to 25.6%, while the “ab” heterozygotes increased from 20.9% to 69.2% over three years. Moreover, β-*tubulin* “aa” homozygous females were significantly more fertile than heterozygotes before treatment (67% versus 37%) and 12 months after the last IVM dose in the groups treated annually (60% versus 17%). No significant selection was observed in the control genes.

## Strengths and Limitations of the Study

A major strength of this study is that the *O. volvulus* parasites were collected from the same individuals before and after IVM treatments. Therefore the observed changes in genotype frequencies between IVM-naïve and treated *O. volvulus* populations are not due to factors such as geographical or sampling effects.

The main limitation of this study was that some worm samples could not be genotyped, thus reducing the number that could be analyzed, particularly after treatment. This limitation might also have impeded the genotyping of DNA, ideally prepared from worm sections instead of just whole females. What is given as a single genotype is, in fact, a consensus of multiple genotypes including the adult female body and progeny (uterine embryos and microfilariae). Furthermore, the samples from unfertile females, which probably represent true singletons, were treated the same as those from females classified as being of low or high fertility. Consequently, the study leaves unanswered questions including whether the selection of the β-*tubulin* heterozygote genotypes is on the females or their progeny. If it is on the progeny, questions remain regarding the fitness and susceptibility to IVM treatment of the β-*tubulin* heterozygote microfilariae once they develop into the infective stage larvae and enter a new human host.

The authors do not present a hypothesis to explain why IVM causes selection for β-*tubulin* heterozygote genotypes. The glutamate-gated chloride channels are thought to be involved in the mode of action of IVM and resistance to the drug [28]. Treatment with IVM is known to cause a loss of polymorphism not only at certain β-*tubulin* gene loci, but also at certain loci of the genes encoding the gamma-aminobutyric acid receptor, glutamate-gated chloride channel, and ATP-binding cassette transporter of IVM-resistant *H. contortus* [23]. It will therefore be important to examine for polymorphisms in these genes in the uniquely collected *O. volvulus* female worms described in this study and in future studies.

Despite these caveats, the study indicates that IVM causes genetic selection on *O. volvulus* worms and points to the daunting possibility of the spread of IVM-resistant parasites in endemic regions that have been treated with IVM.

## Implications of the Study for River Blindness Control

The finding that IVM treatment selected for β-*tubulin* heterozygotes and that this selection was dependent on dosage raises important concerns for the current river blindness control programs. These concerns are heightened by the fact that this gene has been linked with IVM resistance in another parasitic nematode [26], and by the recent evidence that IVM resistance is occurring in *O. volvulus* [17]. Semiannual or more frequent treatments are ongoing in some endemic areas and are under consideration in other areas. Such treatment might increase the selection pressure. Therefore, Bourguinat and colleagues’ study is a wake-up call for control programs to select their treatment regimens carefully and to develop plans for detecting IVM resistance and the associated genetic markers (control programs will require additional funding for these plans). This study presents a possible structure of study design that will incorporate the detection and validation of the genetic markers associated with IVM resistance.

Simultaneously, we need to greatly increase our current level of effort and support to develop and test a new generation of control tools for onchocerciasis. These tools should include both vaccines and macrofilaricides (drugs which kill adult worms) that have new classes of chemistry with novel modes of action. Recent breakthroughs now make macrofilaricide development more feasible, and accordingly such development is now a high-priority goal with the World Health Organization’s Special Programme for Research and Training in Tropical Diseases and the Bill and Melinda Gates Foundation [29,30]. The development of an anti-*Onchocerca* vaccine has been the focus of research supported by the Edna McConnell Clark Foundation [31]. It may be possible to link such a vaccine with drug treatments in a program of vaccine-linked chemotherapy [32,33]. These new generations of control tools would complement the present control measure—the establishment of sustainable community-directed treatment with IVM—and ultimately support the long-term goal of eliminating onchocerciasis as a public health problem in Sub-Saharan Africa.
